# Labeling and Characterization of Human GLP-1-Secreting L-cells in Primary Ileal Organoid Culture

**DOI:** 10.1016/j.celrep.2020.107833

**Published:** 2020-06-30

**Authors:** Deborah A. Goldspink, Van B. Lu, Emily L. Miedzybrodzka, Christopher A. Smith, Rachel E. Foreman, Lawrence J. Billing, Richard G. Kay, Frank Reimann, Fiona M. Gribble

**Affiliations:** 1 Wellcome Trust – MRC Institute of Metabolic Science Metabolic Research Laboratories, Addenbrooke’s Hospital, Hills Road, Cambridge CB2 0QQ, UK

**Keywords:** organoids, GLP-1, L-cells, CRISPR-Cas9, mass spectrometry, RNA sequencing, electrophysiology, calcium, diabetes, obesity

## Abstract

Glucagon-like peptide-1 (GLP-1) from intestinal L-cells stimulates insulin secretion and reduces appetite after food ingestion, and it is the basis for drugs against type-2 diabetes and obesity. Drugs targeting L- and other enteroendocrine cells are under development, with the aim to mimic endocrine effects of gastric bypass surgery, but they are difficult to develop without human L-cell models. Human ileal organoids, engineered by CRISPR-Cas9, express the fluorescent protein Venus in the proglucagon locus, enabling maintenance of live, identifiable human L-cells in culture. Fluorescence-activated cell sorting (FACS)-purified organoid-derived L-cells, analyzed by RNA sequencing (RNA-seq), express hormones, receptors, and ion channels, largely typical of their murine counterparts. L-cells are electrically active and exhibit membrane depolarization and calcium elevations in response to G-protein-coupled receptor ligands. Organoids secrete hormones in response to glucose and other stimuli. The ability to label and maintain human L-cells in organoid culture opens avenues to explore L-cell function and develop drugs targeting the human enteroendocrine system.

## Highlights

• Enteroendocrine differentiation and labeling in human small intestinal organoids
• RNA-seq and peptide LC-MS profiling of purified human GLP-1-secreting L-cells
• Human ileal L-cells are electrically active and respond to nutritional stimuli

## Graphical Abstract

**Figure:**
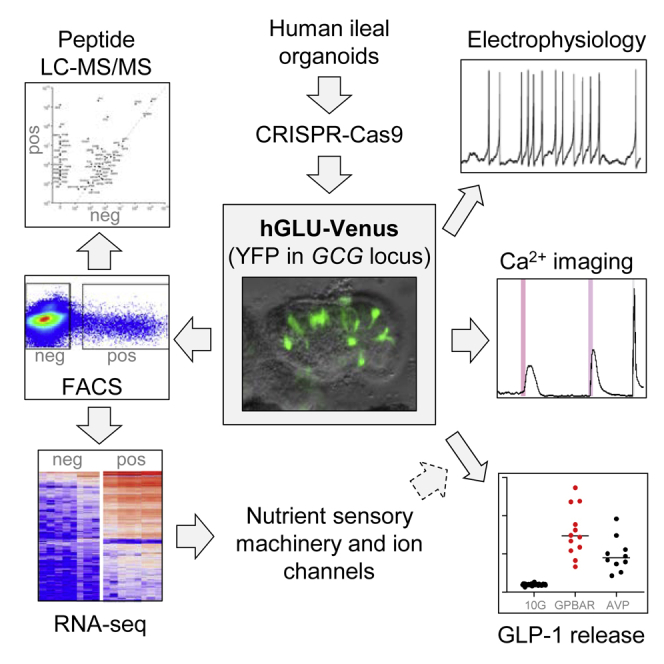

## Teaser

Development of anti-diabetic drugs targeting the hormone-secreting intestinal enteroendocrine system is limited by a lack of human cellular models. Goldspink et al. generate human ileal organoids with fluorescently-labeled GLP-1-secreting L-cells, which respond to nutritional stimuli and are used for transcriptomic, peptidomic, and single-cell functional analyses.

## Introduction

Enteroendocrine cells (EECs) produce gut hormones that regulate energy metabolism and appetite as well as local processes such as nutrient digestion and intestinal motility (Gribble and Reimann, 2019). Glucagon-like peptide-1 (GLP-1), a strong regulator of insulin secretion and appetite (Müller et al., 2019), has been successfully harnessed for the treatment of type 2 diabetes and obesity (Holst, 2020), and a number of drugs linking GLP-1-based peptides with other hormones are in the pipeline, potentially offering superior clinical efficacy with reduced side effects (Capozzi et al., 2018; Frias et al., 2018; Parker et al., 2020). An alternative therapeutic approach under investigation is to stimulate release of the body’s endogenous supplies of gut hormones, thereby mimicking the effects of Roux-en-Y gastric bypass (RYGB) surgery, which results in high post-prandial plasma levels of hormones including GLP-1, peptide YY (PYY), and neurotensin (NTS) as a consequence of rapid nutrient delivery to the distal small intestine and enhanced stimulation of ileal EECs (Gribble and Reimann, 2019). Understanding the physiology and stimulus responsiveness of human GLP-1-secreting L-cells is key to the development of these new classes of therapeutics.

Human EECs, including L-cells, have been notoriously difficult to study because they comprise only ~1% of the intestinal epithelium, and are not identifiable without prior fixation and staining (Sjölund et al., 1983). Our understanding of human EEC physiology is therefore largely restricted to the interpretation of plasma hormone responses to ingested stimuli in different human cohorts, and analyses of intestinal biopsies. By contrast, we now have a relatively good understanding of murine EEC physiology, due to the generation of a range of transgenic mouse models in which EECs express fluorescent labels driven by cell-specific promoters, allowing application of a range of approaches that depend on live cell identification, including transcriptomics, live-cell imaging, and electrophysiology (Gribble and Reimann, 2016). Murine L-cells are electrically active and utilize a variety of sensory proteins for the detection of food ingestion, including sodium-coupled glucose transporters (SGLT1s) for sensing ingested glucose and G-protein-coupled receptors (GPCRs) for detection of many other nutrients (e.g., fatty acids, amino acids) and bile acids (Gribble and Reimann, 2016).

Recent advances have enabled the long-term growth and maintenance of cultured murine and human intestinal epithelium using three-dimensional organoids derived from intestinal crypts or stem cells (Fujii et al., 2018; Sato et al., 2009, 2011). The aim of this study was to use small intestinal organoids for live-cell analysis of human L-cells, by engineering organoids with CRISPR-Cas9 to express a fluorescent protein in the endogenous proglucagon locus. Human L-cells were successfully labeled using this approach, allowing their characterization by RNA sequencing (RNA-seq), electrophysiology, calcium imaging, and their hormone secretory properties.

## Results

**Figure 1:**
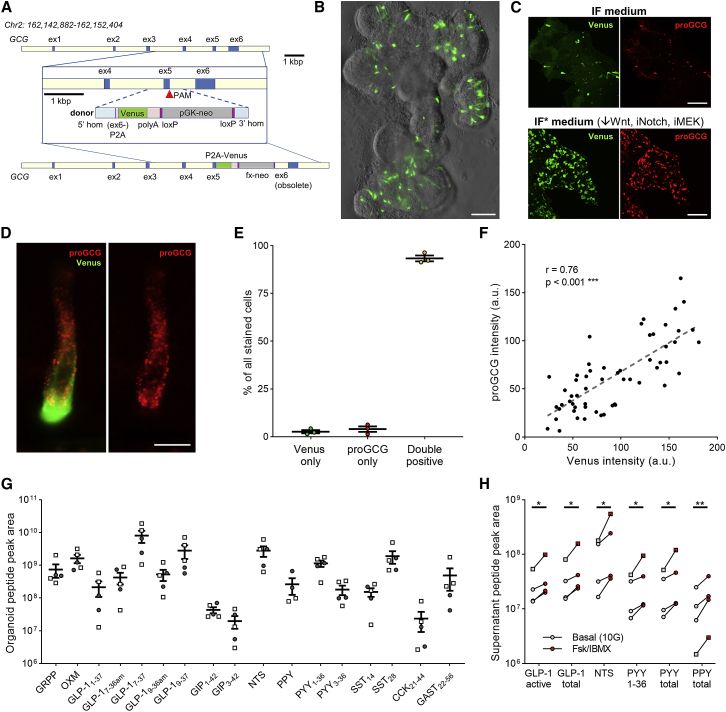
Development of hGLU-Venus Ileal Organoids (A) Schematic representing the components and insertion of the Venus transgene by CRISPR-Cas9. (B) Live image of human hGLU-Venus organoids in IF^∗^ medium. Scale bar, 100 μm. (C) hGLU-Venus organoids grown in IF (above) or IF^∗^ (below) medium were fixed and stained for proglucagon (proGCG, red) and Venus (green). Scale bar, 100 μm. (D) Colocalization of Venus and proglucagon staining in a single Venus positive cell from a fixed hGLU-Venus organoid. Scale bar, 20 μm. (E) Percentage colocalization of Venus and GLP-1 staining, from images obtained as in (F), n = 3. Percentages are given relative to the total number of stained cells. (F) Correlation between fluorescent intensities (in arbitrary units [a.u.]) of staining for Venus and proglucagon in individual cells. (G) Peak areas for different peptides in lysed organoids from LC-MS/MS analysis are depicted from parental organoids (open squares, n = 3) and hGLU-Venus organoids (closed circles, n = 2). (H) Peptide secretion from hGLU-Venus (circles, n = 3) or parental (squares, n = 1) organoids in response to forskolin (fsk) + IBMX (10 μM each), measured in supernatants by LC-MS/MS. ^∗^p < 0.05, ^∗∗^p < 0.01 by paired t test performed on log-transformed data. Mean ± SEM presented in (E) and (G).

Human hGLU-Venus ileal organoids were generated using CRISPR-Cas9-mediated-homology-donor repair to express the yellow fluorescent protein Venus under the control of the proglucagon promoter. A PAM site at the end of exon 5 was targeted to insert the short remaining coding sequence from exon 6, followed by a ribosomal stutter sequence (P2A); Venus; a poly-adenylation sequence; and a PGK-neomycin selection cassette (Figure 1A; STAR Methods). Electroporated organoids were selected in G418 and surviving organoids pooled. Organoids were screened by PCR for correct sequence insertion, and positive organoids were confirmed by direct sequencing of the recombined area. In parallel, we optimized conditions for culturing human organoids to generate functional EECs. Growth of hGLU-Venus organoids in IF medium (Fujii et al., 2018; STAR Methods), or modified medium (IF^∗^) containing inhibitors of mitogen activated protein kinase kinases (MEK1/2) and Notch (Basak et al., 2017; Beumer et al., 2018) with reduced Wnt3A to enhance EEC development, resulted in the appearance of scattered yellow-fluorescent cells that morphologically resembled EECs, with processes extending across the epithelial layer (Figure 1B). Immuno-staining confirmed the dual labeling of cells with antibodies against GFP (detecting Venus) and proglucagon (Figure 1D), with >90% of stained cells being positive for both proglucagon and Venus (Figure 1E). Across individual cells, the intensities of Venus and proglucagon staining were positively correlated, as predicted since Venus expression was driven by the proglucagon promoter in this line (Figure 1F).

Both parental and CRISPR-modified ileal organoids were analyzed by liquid chromatography-tandem mass spectrometry (LC-MS/MS) peptidomics to characterize hormone biosynthesis and secretion. In organoid lysates we detected a range of known gut peptides, including known intestinally processed forms of GLP-1, PYY, NTS, and somatostatin (SST) (Figure 1G). LC-MS/MS analysis of supernatants from organoid cultures treated with or without forskolin plus isobutylmethylxanthine (IBMX) to stimulate secretion, revealed cyclic AMP (cAMP)-dependent secretion of gut hormones known to be released from ileum, including GLP-1, PYY and NTS, as well as PPY (Figure 1H), confirming that EECs in these cultures exhibit functional stimulated secretion. Peptide biosynthesis and secretion were similar between the parental and CRISPR-modified organoid lines.

**Figure 2:**
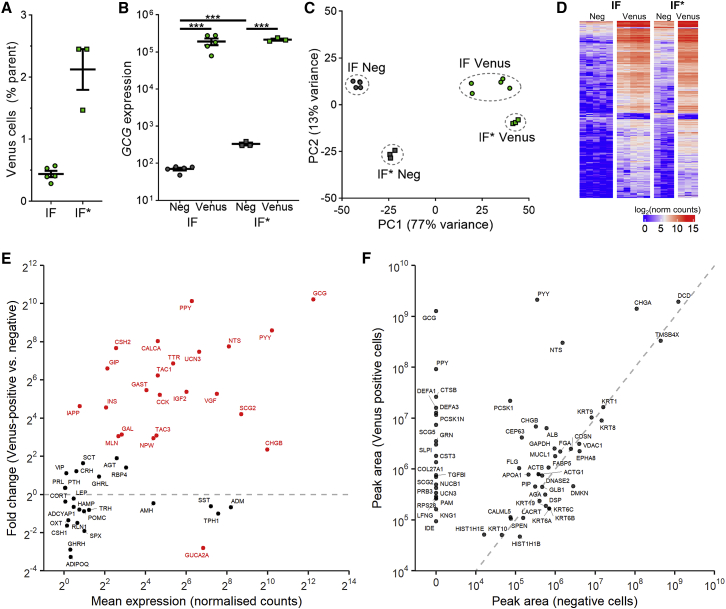
RNA-Seq and LC-MS/MS Peptide Analysis of Cell Populations Purified by Flow Cytometry Fluorescent cells from hGLU-Venus ileal organoids, grown in either IF (n = 5) or IF^∗^ (n = 3) media, were sorted by flow cytometry, and analyzed by RNA sequencing (RNA-seq). (A) Percent of Venus-fluorescent cells compared with parental gate (all live cells, excluding debris, DAPI-positive cells and DRAQ5 negative events). (B) Differential GCG expression across cell populations. ^∗∗∗^p < 0.001, by Wald test. (C) Principal-component analysis of differentially expressed genes between L-cells and non-L-cells. (D) Heatmap showing top 500 differentially expressed genes between L-cells and non-L-cells. (E) RNA-seq-derived expression of genes encoding hormones and vesicular peptides, depicted as the enrichment in the combined L-cell versus non-L-cell populations plotted against the mean expression level, where genes labeled in red were differentially expressed between populations. Differentially expressed genes defined as p <0.05, by Wald test. (F) LC-MS/MS analysis of peptides (combined per parental protein) identified in FACS-sorted Venus-positive cells compared with negative cells, using a false discovery rate of 0.4%. Peptides are labeled by their gene names to avoid confusion. Mean ± SEM presented in (A) and (B).

Venus-fluorescent and non-fluorescent cells were purified by fluorescence-activated cell sorting (FACS) from organoids cultured in either IF medium (n = 5) or IF^∗^ medium (n = 3), for RNA-seq (Figure S1). By FACS analysis, fluorescent cells comprised 0.4% ± 0.1% (mean ± SD, n = 5) of the total cell count in IF medium, and 2.1% ± 0.6% (n = 3) of cells in IF^∗^ medium (Figure 2A). From the RNA-seq data, Venus-positive cells were strongly enriched for the *GCG* message, which was found at ~1000-fold-higher levels in fluorescent compared with non-fluorescent cells (Figure 2B). We will hereafter refer to these Venus-positive cells as *L-cells*. Principal-component analysis (PCA) showed wide separation of L-cells from non-L-cells on the first component (77% of variance), and narrow separation between culture media on the second component (13% of variance; Figure 2C). These distinctions are also evident in the heatmap of the top 500 differentially expressed genes (Figure 2D).

RNA-seq identified enriched expression in L-cells of a number of known ileal gut hormones in addition to *GCG*, including *PYY*, *NTS*, and *PPY*, together with lower levels of hormones typically found more proximally, such as *CCK*, *GIP*, and *GAST* (Figure 2E). Interestingly, L-cells exhibited enriched expression of *TAC1*, a gene normally attributed to enterochromaffin (EC) cells, but whereas *TPH1*, the EC-cell-defining enzyme, was detected in the L-cell population, it was found at similar levels in non-fluorescent cells. Fluorescent cells were FAC-sorted separately for LC-MS/MS peptidomic analysis (Figure 2F), confirming that they contained high and enriched levels of gut hormones from proglucagon (GCG), PYY, PPY, and NTS, as well as detectable levels of UCN3 and several secretogranin/chromogranin-derived peptides.

**Figure 3:**
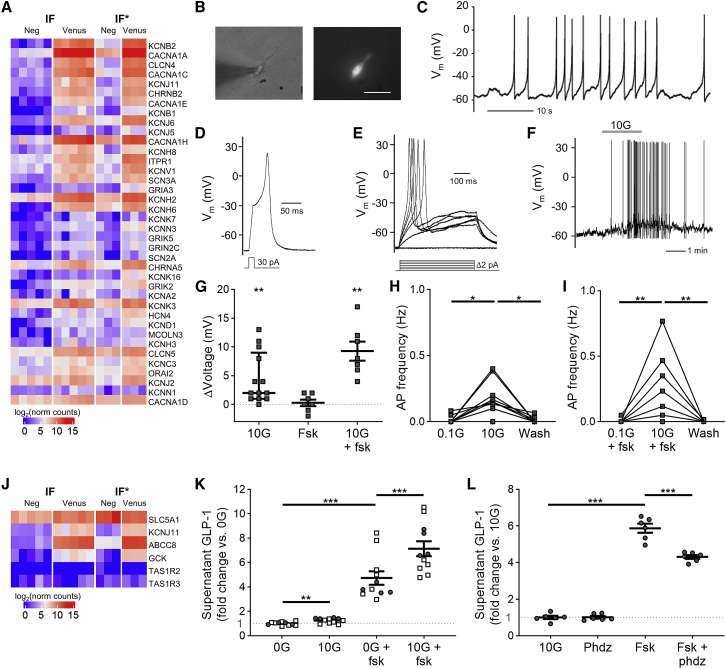
Electrical Activity and Glucose Sensing (A) RNA-seq analysis of differentially expressed ion channels in sorted cells from hGLU-Venus ileal organoids, grown in either IF or IF^∗^ media. Genes included had p <0.05, by Wald test, and expression level >2-fold enriched in the combined Venus-positive cell populations. (B) Images of Venus-positive cell studied by electrophysiology. L-cells were identified by their yellow fluorescence (right) and patched using phase contrast (left). Scale bar, 50 μm. (C) Perforated-patch, whole-cell current-clamp recording of a Venus-positive cell exhibiting spontaneous electrical activity. (D) Perforated-patch recording of Venus-positive cell held at −75 mV, with action potential evoked by a 10-ms depolarizing current injection of 30 pA, as indicated by the pulse protocol below. (E) As in (D), with longer (500-ms) current injection pulses, as shown in the pulse protocol below. (F) Venus-positive cell recorded as in (C), with elevation of the glucose concentration from 0.1 to 10 mM for the period indicated by the horizontal bar labelled 10G. (G) Change in membrane potential for cells recorded as in (F), on addition of 10 mM of glucose (10G), 10 μM of forskolin (fsk), or 10 mM glucose in the presence of 10 μM fsk (10G+fsk). Inter-spike membrane potential was used for cells that were firing action potentials. ^∗∗^p < 0.01, by one-sample t test, n = 7–13. (H) Mean action potential frequencies for cells recorded in 0.1, 10, and 0.1 mM of glucose. (I) As in (H), but cells started in 0.1 mM of glucose with 10 μM fsk (0.1G+fsk) were then perfused with solution containing 10 mM glucose in forskolin (10G+fsk), and finally washed out with 0.1 mM glucose. (H and I) ^∗^p < 0.05, ^∗∗^p < 0.01, by Kruskal-Wallis with Dunn’s multiple comparisons test; n = 13 (H) and n = 7 (I). (J) Heatmap showing expression of candidate glucose sensing machinery by RNA-seq. (K) GLP-1 secretion from human organoid cultures in absence and presence of 10 mM glucose (10G) or 10 μM fsk, as indicated. Control solutions contained zero glucose (0G). n = 11 from n = 5 experiments, performed with either the parental organoids (open squares) or the hGLU-Venus (closed circles) organoids. (L) Sensitivity of glucose-triggered GLP-1 release to phloridzin (Phdz, 5 μM), in the absence or the presence of 10 μM fsk (n = 6 from n = 3 experiments). Statistical comparisons in (K) and (L) were performed by three-way ANOVA, including experimental replicate as a parameter, with Tukey’s honest significant difference test. ^∗^p < 0.05, ^∗∗^p < 0.01, ^∗∗∗^p < 0.001. Mean ± SEM presented except for (G); median ± interquartile range are presented.

From the RNA-seq analysis, we identified differential expression in L-cells of a number of voltage-gated ion channels (Figure 3A), including *SCN3A* (Na_v_1.3), *CACNA1A* (P/Q type Ca^2+^ channel, Ca_v_2.1), and *KCNB2* (K_v_2.2). L-cells also expressed the L-type Ca^2+^ channel subunit *CACNA1C* (Ca_v_1.2), widely implicated in Ca^2+^-dependent vesicular exocytosis (Gilon et al., 2014), as well as T-type (*CACNA1H*) Ca^2+^ channels. To perform electrophysiological recordings from fluorescent cells, organoids from IF^∗^ media were disrupted and plated onto Matrigel-coated dishes (Figure 3B). Perforated-patch recordings from fluorescent cells revealed that 20/52 cells exhibited spontaneous action potential firing (Figure 3C). Across all L-cells studied, the mean inter-spike (or resting) membrane potential was −60 ± 1.6 mV. All fluorescent cells exhibited evoked action potentials in response to current injection (Figures 3D and 3E). The mean action potential threshold was −39.2 ± 1 mV (n = 52), the mean action potential peak was +25.2 ± 0.9 mV, and the mean action potential half width was 22.1 ± 0.9 ms.

In electrophysiological recordings, glucose triggered membrane depolarization and action potential firing (Figures 3F–3I). Examination of the RNA-seq data for potential glucose-sensing machinery (Figure 3J) revealed that hGLU-Venus L-cells exhibited high and selective expression of the K_ATP_ channel subunits *KCNJ11/ABCC8* and glucokinase (*GCK*), as well as high levels of *SLC5A1* (encoding SGLT1). Expression of the two subunits making up the sweet taste receptor, *TAS1R2* and *TAS1R3*, was barely detectable. GLP-1 secretion from organoid cultures was stimulated by 10 mM glucose in the absence or the presence of 10 μM forskolin (Figure 3K), and impaired by the SGLT1 inhibitor phloridzin (5 μM; Figure 3L), suggesting the involvement of an SGLT1-dependent glucose-sensing pathway underlying GLP-1 release from organoid-derived human L-cells.

**Figure 4:**
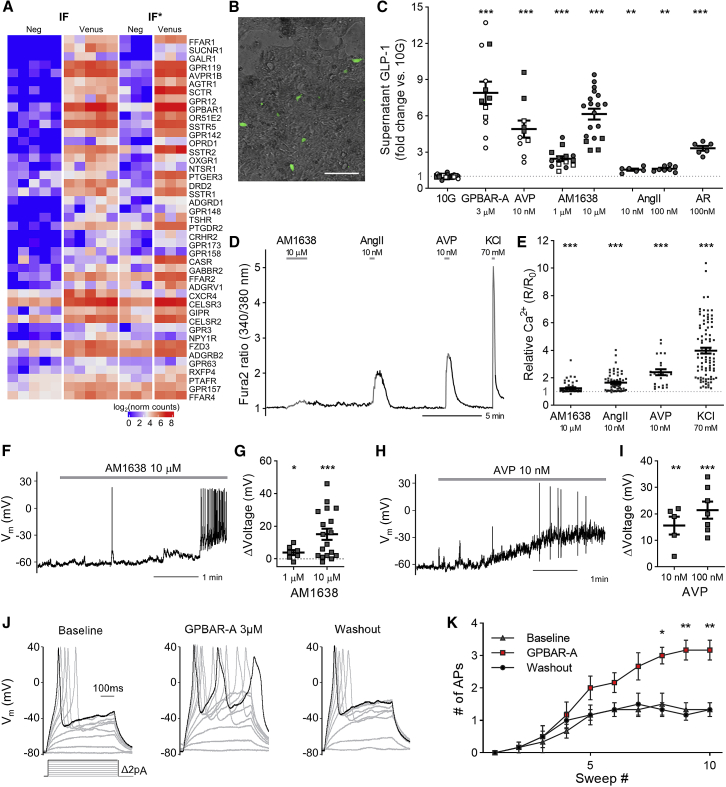
Stimulus Detection by GPCRs (A) RNA-seq analysis of differentially expressed GPCRs in sorted cells from hGLU-Venus ileal organoids, grown in either IF or IF^∗^ media. Genes included had p <0.05, by Wald test, and expression level >2-fold enriched in the combined Venus-positive cell populations. Scale bar, 100 μm. (B) Image of hGLU-Venus organoids plated in 2D for secretion and imaging. (C) GLP-1 release from organoids in response to a variety of GPCR agonists. All solutions contained 10 mM of glucose (10G), with additions of GPBAR-A (GPBAR1 agonist), AVP, AM1638 (FFAR1 agonist), AngII (angiotensin II), and AR (AR231453, GPR119 agonist) as indicated. n = 6–19 replicates each from three to eight experiments; open and filled symbols indicate results from parental and hGLU-Venus organoids, respectively; circles and squares indicate results from IF and IF^∗^ cultures, respectively. Statistical comparisons were performed by two-way ANOVA, including experimental replicate as an independent variable, with Tukey’s honest significant difference test. (D) Fura2 ratiometric calcium recording from a single Venus-positive-cell, perfused with stimuli as indicated. (E) Data from multiple cells recorded as in (D), expressed as R/R_0_ (fura2 ratio in test solution/fura2 ratio in basal solution). Number of responsive cells (defined as 10% increase in maximal Fura2 ratio from baseline) shown. ^∗∗∗^p < 0.001 by one-sample Wilcoxon test. (F) Perforated-patch, current-clamp recording of a Venus-positive cell responsive to the FFAR1 agonist, AM1638 (10 μM). (G) Change in membrane potential triggered by AM1638 (1 or 10 μM) for cells recorded as in (F). ^∗^p < 0.05, ^∗∗∗^p < 0.001, by one-sample t test. (H) Perforated-patch, current-clamp recording of a Venus-positive cell responsive to AVP (10 nM). (I) Change in membrane potential triggered by AVP (10 or 100 nM) for cells recorded as in (H). ^∗∗^p < 0.01, ^∗∗∗^p < 0.001, by one-sample t test. (J) Evoked action potentials at baseline (left) and during application of GPBAR1 agonist (3 μM, middle), and following washout (right). (K) Mean number of action potentials evoked during the 500-ms current pulses, recorded as in (J). n = 6. Statistical significance determined using the Holm-Sidak method, showing adjusted p values: ^∗^p < 0.05, ^∗∗^p < 0.01, comparing baseline and GPBAR-A treatment for each current injection step. Mean ± SEM presented in graphs with individual data-point values.

We next examined the RNA-seq data for expression of GPCRs in human L-cells (Figure 4A). Many receptors previously implicated in post-prandial gut hormone secretion were highly and selectively expressed in ileal-organoid-derived L-cells, including *FFAR1*, *GPBAR1*, *GPR119*, *AGTR1*, and *AVPR1B*. Confirming the functional activity of these receptors, GLP-1 secretion from human organoids plated in two-dimensional cultures (Figure 4B) was increased by AM1638 (FFAR1 agonist, 1 μM and 10 μM), arginine vasopressin (AVP) (10 nM), angiotensin II (AngII; 10 nM), AR231453 (GPR119 agonist, 100 nM), and a GPBAR1 agonist (3 μM; Figure 4C). For receptors known to be coupled to the G-protein G_q_ (FFAR1, AVPR1B, and AGTR1), we examined intracellular Ca^2+^ responses by fura2 ratiometric imaging in two-dimensional cultures (Figures 4D and 4E). Ca^2+^ elevation (defined as a fold-change in fura2 ratio of >10%) was seen in all L-cells (91/91) following application of 70 mM of KCl, 24/24 cells in response to AVP (10 nM), 25/56 cells in response to the FFAR1 agonist AM1638 (10 μM), and 46/62 cells in response to AngII (10 nM). In electrophysiological recordings, the G_q_-coupled-receptor agonists AVP and AM1638 triggered membrane depolarization and action potential firing (Figures 4F–4I). Stimulation of the G_s_-coupled receptor GPBAR1 increased the capacity of cells to fire evoked action potentials (Figures 4J and 4K).

## Discussion

The development of hGLU-Venus ileal organoids, together with protocols to maintain functional EECs in organoid cultures, has provided the opportunity to study the single-cell properties of human GLP-1-secreting L-cells. The properties of human L-cells identified with this method fit with published literature describing the physiology of GLP-1 release in humans subjects, and the transcriptome of human L-cells obtained after cell fixation (Roberts et al., 2019). We show here that human L-cells are electrically active, express a repertoire of hormones matching previous immunostaining data, and secrete GLP-1 in response to ligands of a variety of nutrient- and hormone-sensing GPCRs.

The optimized protocols for generating EECs in human organoid cultures facilitated screening for CRISPR-Cas9-modified organoids, as the appearance of fluorescent EEC-like structures could be used as an additional marker for organoids expressing the inserted promoter-less reporter construct. We used two different media for promoting EEC development: in IF medium, the L-cell count was relatively low, mirroring the low frequency of L-cells in native intestinal epithelium (<1%). In IF^∗^ medium, fluorescent cell numbers were higher and above physiological levels. However, the RNA-seq analyses of L-cells from both media were similar.

Human ileal organoids faithfully generated and processed a range of gut peptides, including GLP-1, NTS, and PYY, and no differences in peptide content or secretion were observed in GLU-Venus compared with parental organoid lines. All L-cell peptides were secreted in response to a test stimulus of forskolin + IBMX, as demonstrated by LC-MS/MS, and immunoassay-detected GLP-1 was secreted in an expected pattern in response to a range of GPCR agonists that have previously been implicated in stimulating GLP-1 release in a range of model systems (Gribble and Reimann, 2016).

Glucose triggered membrane depolarization and action potential firing in human L-cells, and stimulated GLP-1 release in the presence of forskolin that was impaired by phloridzin, suggesting an involvement of SGLT1, as found previously in mouse and rat (Gorboulev et al., 2012; Kuhre et al., 2015). Glucose is a good stimulus of GLP-1 release in human subjects, as readily demonstrated by the large plasma GLP-1 responses to an oral glucose challenge in patients following gastrectomy surgery with Roux-en-Y gastric reconstruction, where ingested glucose passes rapidly to the lower small intestine (Roberts et al., 2018). The likely involvement of SGLT1 in human L-cell glucose sensing is supported by a number of observations, including that the non-metabolizable glucose analog 3-O-methylglucose was similarly effective to glucose in triggering GLP-1 release following oral ingestion (Wu et al., 2012), and that phloridzin inhibited glucose-triggered GLP-1 secretion from human small intestinal tissue biopsies (Sun et al., 2017). Although K_ATP_ channels and glucokinase are expressed in human L-cells, as also demonstrated previously by immunostaining (Nielsen et al., 2007), there is little evidence that they contribute to L-cell sensing of ingested glucose. Our finding of low/undetectable expression of sweet taste receptor subunits in human L-cells also matches a number of reports that sweet tastants are poor stimuli of GLP-1 release in human subjects (Ma et al., 2009; Wu et al., 2012).

This study demonstrates that human L-cells are electrically active, firing spontaneous action potentials, and exhibiting membrane depolarization in response to nutrient stimuli. While we have not attempted here to dissect which voltage-gated ion channels underlie the electrical activity, the action potential threshold and waveform are similar to those found in murine L-cells (Goldspink et al., 2018; Rogers et al., 2011). We identified high expression of the voltage-gated sodium channel Na_v_1.3, as well as P/Q-, L-, and T-type Ca^2+^ channels; this is consistent with previous murine data, and with the finding that GLP-1 release from human intestinal biopsies was blocked by the L-type Ca^2+^-channel-inhibitor nifedipine (Sun et al., 2017).

GLP-1 secretion from human ileal organoids was triggered by agonists of FFAR1, GPR119, AGTR1, AVPR1B, and GPBAR1, mirroring similar data obtained previously using murine tissue and organoids (Goldspink et al., 2018; Pais et al., 2016a, 2016b), but contrasting with human primary colonic cultures that we previously reported to secrete GLP-1 in response to GPBAR1 and FFAR1, but not GPR119 agonism, albeit using different ligands and concentration ranges (Habib et al., 2013). In hGLU-Venus cells, agonists of the G_q_-coupled receptors FFAR1 and AVPR1B triggered membrane depolarization, and GPBAR1 agonist enhanced the capacity for membrane potential firing, as also observed previously in murine L-cells (Goldspink et al., 2018). Whereas FFAR1 has been linked to enteroendocrine secretion via the TRP channels TRPC3 in mouse primary L-cells (Gribble et al., 2017) and TRPM5 in the STC-1 cell line (Shah et al., 2012), neither of these channels exhibited differential mRNA expression in human organoid-derived L-cells; further mechanistic studies will be required to identify whether the molecular machinery linking GPCR activation to hormone secretion is similar in human compared with murine L-cells.

### Conclusion

Organoid cultures support the generation of fully functional human L-cells, and together with CRISPR-Cas9 labeling, enable identification of live human L-cells for single-cell and transcriptomic analysis. Human L-cells exhibited properties very similar to their murine counterparts across a wide range of parameters, including hormone biosynthesis and processing, electrical activity, and responsiveness to nutrient, hormonal, and bile-acid stimuli. Application of the method to organoids from other regions of the human GI tract, combined with the harnessing of different gut hormonal promoters, will enable characterization of the full range of human EEC types, and facilitate screening for drugs that increase gut hormone secretion for the treatment of metabolic diseases.

## STAR★Methods

### Key Resources Table

**Table:** 

REAGENT or RESOURCE	SOURCE	IDENTIFIER
**Antibodies**		

Alexa Fluor donkey anti-goat 488	Invitrogen	A11055; RRID: AB_2534102
Alexa Fluor donkey anti-mouse 555	Invitrogen	A31570; RRID: AB_2536180
Alexa Fluor donkey anti-rabbit 647	Invitrogen	A31573; RRID: AB_2536183
Goat anti-GFP	Abcam	ab5450; RRID: AB_304897
Mouse anti-proglucagon	Santa Cruz	sc-514592; RRID: AB_2629431
Rabbit anti-chromogranin A	Santa Cruz	sc-13090; RRID: AB_2080982

**Bacterial and Virus Strains**		

One Shot OmniMAX 2 T1R Chemically Competent *E. coli*	ThermoFisher Scientific	C854003

**Biological Samples**		

Human ileum	Addenbrooke’s Hospital Human Research Tissue Bank	N/A

**Chemicals, Peptides, and Recombinant Proteins**		

[Leu^15^]-gastrin I (human)	Sigma-Aldrich	G9145
A-83-01	Tocris	2939
AM-1638 (FFAR1 agonist)	Generon	HY-13467
Amphotericin B	Sigma-Aldrich	A4888
Angiotensin II (AngII)	Sigma-Aldrich	A9525
AR231453 (GPR119 agonist)	Sigma-Aldrich	SML1883
Arginine vasopressin acetate (AVP)	Sigma-Aldrich	V9879
B27 supplement (minus vitamin A)	Invitrogen	12587-010
BTXpress electroporation solution	BTX	45-0805
DAPT	Generon	1855
EGF (murine)	Invitrogen	PMG8043
FGF-2/basic (human)	Peprotech	AF-100-18C
Forskolin	Sigma-Aldrich	F6886
Fura2-AM	Invitrogen	F1221
G418 (geneticin)	Sigma-Aldrich	A1720
GPBAR-A	Sigma-Aldrich	SML1207
IBMX (3-isobutyl-1-methylxanthine)	Sigma-Aldrich	I7018
IGF-1 (human)	Biolegend	711308
N2 supplement	Invitrogen	17502-048
NAC (N-Acetyl-L-Cysteine)	Sigma-Aldrich	A9165
Nicotinamide	Sigma-Aldrich	N0636
Noggin (murine)	Peprotech	250-38
PD0325901	Sigma-Aldrich	PZ0162
Phloridzin dihydrate	Sigma-Aldrich	P3449
Rock Inhibitor Y27632	Tocris	1254
SB202190	Sigma-Aldrich	S7067
Y-27632 dihydrochloride	Tocris	1254

**Critical Commercial Assays**		

Total GLP-1 (ver. 2) Assay	MesoScale Discovery	K150JVC
SMARTer Stranded Total RNA-Seq v2 Pico Input Mammalian Kit	Takara Bio	634413
Oasis HLB μElution Solid Phase Extraction (SPE) Plate	Waters	186008052

**Deposited Data**		

Bulk RNA sequencing data	Gene expression omnibus https://www.ncbi.nlm.nih.gov/geo/	GSE148224
Sorted cell peptidomics data	PRIDE repository https://www.ebi.ac.uk/pride/	PXD017825

**Experimental Models: Cell Lines**		

hGLU-Venus ileal organoid line	This manuscript	
L-Wnt3A cell line	ATCC	CRL-2647; RRID: CVCL_0635
R-spondin1 cells	Trevigen	3710-001-K

**Oligonucleotides**		

Forward primer to check 5′ integration of Venus insert CTCTTGACGATATTTTGCAGTGT	This paper	N/A
Reverse primer to check 5′ integration of Venus insert ATCAGCTTCAGGGTCAGCTT	This paper	N/A
Forward primer to check 3′ integration of Venus insert TGGCTACCCGTGATATTGCT	This paper	N/A
Reverse primer to check 3′ integration of Venus insert CCCTTTGTCCATAAATCCCTCC	This paper	N/A

**Recombinant DNA**		

pTOPO_hGLU_P2A_Venus_PA_fxPGKNeo_hGLU	This paper	N/A
px458-pSpCas9(BB)-2A-GFP CRISPR-Cas9	Ran et al., 2013	Addgene #48138

**Software and Algorithms**		

BBMap (v38.76)	Bushnell B.	http://sourceforge.net/projects/bbmap/
BD FACSChorus	BD Biosciences	N/A
cutadapt (v2.7)	Martin, 2011	https://cutadapt.readthedocs.io/en/stable/
DESeq2 (v1.24.0)	Love et al., 2014	https://bioconductor.org/packages/release/bioc/html/DESeq2.html
featureCounts (v2.0.0)	Liao et al., 2014	http://subread.sourceforge.net/
Fiji	Schindelin et al., 2012	https://imagej.net/Fiji
Metafluor	Molecular Devices	N/A
pCLAMP (v.7.3)	Molecular Devices	N/A
PEAKS (v8.5)	Bioinformatics Solutions Inc	http://www.bioinfor.com/peaks-studio/
RStudio (v1.2)		https://www.rstudio.com/products/rstudio
Xcalibur (v3.0.63)	ThermoFisher Scientific	https://www.thermofisher.com/order/catalog/product/OPTON-30487
ZEN 3.1 (blue edition)	ZEISS	https://www.zeiss.com/microscopy/int/products/microscope-software/zen.html

### Resource Availability

#### Lead Contact

Further information and requests for resources and reagents should be directed to and will be fulfilled by the Lead Contact, Fiona Gribble (fmg23@cam.ac.uk).

#### Materials Availability

The hGLU-Venus human organoid line generated in this study will be made available on request, subject to ethical restrictions and Material Transfer Agreements.

#### Data and Code Availability

Bulk RNA sequencing data are available in the NCBI GEO repository (GSE148224). The sorted cell peptidomics data have been deposited to the ProteomeXchange Consortium via the PRIDE partner repository (PXD017825).

### Experimental Model and Subject Details

#### Organoid culture media and enteroendocrine differentiation

Two base organoid media based on previous reports, termed H-WENR (Sato et al., 2011) and IFE (Fujii et al., 2018), were used. Both media contain the following components in Advanced DMEM:F12 (ADF, Invitrogen): 80% Wnt3A conditioned media, 10% RSPO1 conditioned media, 50 ng/ml murine EGF (Invitrogen), 100 ng/ml murine noggin (BMP-4 antagonist, Peprotech), N2 supplement (Invitrogen), B27 supplement (without vitamin A, Invitrogen), 1 mM N-acetyl-L-cysteine (NAC, Sigma-Aldrich), 500 nM A83-01 (Tocris), 10 nM human [Leu^15^]-gastrin I (Sigma-Aldrich), 2 mM L-glutamine (Sigma-Aldrich), 10 μM Y-27632 (Tocris) and an additional 50 units/ml penicillin (Sigma-Aldrich) and 50 μg/ml streptomycin (Sigma-Aldrich). H-WENR medium also contains 10 nM p38 inhibitor SB202190 (Sigma-Aldrich) and 10 mM nicotinamide (Sigma-Aldrich), whereas IFE medium contains 100 ng/ml recombinant human insulin-like growth factor 1 (IGF-1, Biolegend) and 50 ng/ml recombinant human fibroblast growth factor 2 (FGF-2 basic, Peprotech). For successful enteroendocrine lineage differentiation, organoids were grown in IFE medium until large organoids had formed (14-21 days) before EGF was removed from cultures (termed IF media), which led to appearance of fluorescent cells in hGLU-Venus organoids over the next 10-14 days. For accelerated enteroendocrine differentiation, Wnt3A conditioned media was reduced to 10% in IF medium and 10 μM Notch inhibitor DAPT (Generon) and 100 nM MEK inhibitor PD0325901 (Sigma-Aldrich) were added to the culture (termed IF^∗^ media), which resulted in enhanced appearance of fluorescent cells after 3-5 days.

Conditioned Wnt3A medium was generated using the L-Wnt3A cell line (ATCC® CRL-2647) cultured at 37°C (5% CO_2_) in DMEM (4500 mg/l glucose, GIBCO), supplemented with 4 mM L-glutamine, 10% fetal bovine serum (FBS, GIBCO), 100 units/ml penicillin, 100 μg/ml streptomycin, 0.4 mg/ml of G418 selection antibiotic (Sigma-Aldrich), and passaged twice a week. G418 was omitted from cultures for Wnt3A conditioned medium, with collection taken at day 4 and day 7. RSPO1 conditioned medium was generated from Cultrex® R-spondin1 cells (Trevigen) using an adapted protocol (Fujii et al., 2015). Cells were cultured at 37°C (5% CO_2_) in DMEM (4500 mg/l glucose) supplemented with 10% FBS, 100 units/ml penicillin, 100 μg/ml streptomycin, 1% GlutaMAX (GIBCO) and 0.3 mg/ml of zeocin selection antibiotic (GIBCO), with cells passaged twice per week. Zeocin was omitted from culture for 3 days followed by confluent re-seeding in ADF supplemented with 100 units/ml penicillin, 100 μg/ml streptomycin, 1 mM HEPES and 1% GlutaMAX before collection of RSPO1 conditioned media at day 7. After collection, conditioned media were filter sterilized, frozen at −20°C and used within 6 months.

#### Organoid establishment, maintenance, and cryopreservation

Human ileal organoid lines were generated from anonymized surgical specimens obtained from Tissue Bank at Addenbrooke’s Hospital (Cambridge, UK); ethical approval was granted by the East of England – Cambridge Central Research Ethics Committee (ref: 09/H0308/24). Organoids were generated using a modified protocol (Dye et al., 2019; Goldspink et al., 2018; Sato et al., 2011). Fresh ileal surgical specimens were chopped into 2-3 mm^2^ pieces and washed in ice cold PBS. Tissue pieces were treated with 30 mM EDTA for 3 × 10 min, with tissue shaken in PBS following each EDTA treatment. The fraction with isolated crypts and no villi was then washed in PBS. As fresh intact isolated ileal crypts do not proliferate well in organoid culture, isolated crypts were digested with TrypLE Express (GIBCO) for 3 min at 37°C to generate small cell clusters. Cell clusters were resuspended in ice cold basement membrane extract (BME, Cultrex PathClear Reduced Growth Factor Type 2), which was polymerized at 37°C for 30-60 min as small domes (15-20 μl) in 6- or 48-well plates. H-WENR or IFE organoid media was then overlaid to cover the domes and changed three times per week.

Organoids were passaged in a 1:5-1:10 split after 14-21 days, once they had formed large budded structures. TrypLE was added to multiwell plates and organoids were collected into a 15ml centrifuge tube. Following digestion at 37°C for 3-10 min until organoids begin to break open and clump, organoids were centrifuged (400 g, 4 min). Supernatant was removed and organoids were mechanical sheared with a P200 micropipette and then reseeded into fresh BME and cultured as before.

Human intestinal organoids were routinely grown for up to 12 months. Intact mature budded organoids were cryopreserved in freezing medium (45% ADF, 45% heat-inactivated FBS and 10% DMSO supplemented with 10 μM Y-27632). Frozen organoids were defrosted and then digested in TrypLE for 2 min at 37°C before reseeding small clusters in BME as above.

#### hGLU-Venus reporter line generation

A CRISPR site (TTCAGACCAAAATCACTGAC**AGG**) at the end of exon 5 in the proglucagon (*GCG)* gene was targeted. The donor was originally generated using gBlocks Gene Fragments (Integrated DNA Technologies) with a left homology arm (501bp, with a silent mutation of PAM sequence AGG to AGA) followed by insertion of 3 bases (AAA, last coding amino acid, lysine) from exon 6 (just upstream of *GCG* stop codon), a P2A ribosomal stutter sequence, Venus, a bovine growth hormone polyA sequence and a right homology arm (349bp), this was cloned into the Zero Blunt TOPO PCR system (Invitrogen). As initial electroporation experiments showed very low knock-in efficiency, a neomycin resistance cassette (loxP-pGK-neo-loxP) was added to the donor 5′ of the right homology arm using Gibson cloning (NEB) to enable antibiotic selection. The target guide was cloned into px458-pSpCas9(BB)-2A-GFP CRISPR-Cas9, a gift from Feng Zhang (Addgene plasmid #48138), using previously published protocols (Ran et al., 2013). Donor and guide plasmids were confirmed by Sanger sequencing (Source Bioscience), amplified and then purified using a HiSpeed Maxi Kit (QIAGEN). Isolated DNA was concentrated (> 2 μg/μl) by ethanol precipitation and diluted in water.

Electroporation of organoids was performed using previously reported protocols (Fujii et al., 2015). Briefly, ~14 day old ileal organoid cultures grown in H-WENR were extracted from BME using ice cold ADF and then transferred to TrypLE (supplemented with 10 μM Y-27632) and digested at 37°C for 40 min, mechanically dissociating every 10 min with a P1000 pipette. Digested 1-10 cell clusters were washed twice with Opti-MEM (Invitrogen, supplemented with 10 μM Y-27632) and passed through a 20 μm cell strainer to remove any remaining large cell clusters. 36 μg of donor vector and 27 μg of CRISPR guide plasmid were used for transfection of 0.6 × 10^6^ cells using a NEPA21 Type II electroporator (NEPAGENE) as previously described. Antibiotic selection with 0.5 mg/ml G418 was started 5 days post-electroporation and continued for 3 months. Only 10 organoids (< 1 per well) survived selection and were pooled together to generate the hGLU-Venus line, as in our hands human ileal organoids poorly tolerated colony picking. DNA was extracted using QuickExtract DNA Extraction Solution (Lucigen), with successful integration tested by PCR screening. The 5′ integration site was checked with a forward primer (CTCTTGACGATATTTTGCAGTGT) upstream of left arm matched with a reverse primer in Venus (ATCAGCTTCAGGGTCAGCTT) and the 3′ integration site was checked with a forward primer in neo (TGGCTACCCGTGATATTGCT) with a reverse primer outside the right homology arm (CCCTTTGTCCATAAATCCCTCC). Sanger sequencing confirmed successful integration. This hGLU-Venus line has been maintained for more than 12 months at a time and has been cryopreserved and re-established from frozen stocks several times.

### Method Details

#### Immunohistochemistry and imaging of live organoids

Differentiated organoids were fixed and stained as previously described (Goldspink et al., 2017). Briefly, organoids were fixed in 4% paraformaldehyde (PFA) for 20 min, washed 3 × 10 min (PBS with 0.1% Triton x-100, Sigma-Aldrich) and then antigen-retrieval performed using sodium citrate pH 6.0 for 2 × 20 min at 80°C. Organoids were blocked in secondary antibody host serum for 1 hour and then incubated overnight at 4°C with primary antibodies – mouse anti-proGCG (1/50, Santa Cruz sc-514592), goat anti-GFP/Venus (1/500, Abcam ab5450) and rabbit anti-CHGA (1/200, Santa Cruz sc-13090). Donkey secondary antibodies (all 1/300 Alexa Fluor, Invitrogen) were incubated for two hours at room temperature: anti-mouse 555 (A31570), anti-goat 488 (A11055) and anti-rabbit 647 (A31573) were used. Stained organoids were mounted on slides in Hydromount (National Diagnostics) and imaged on an SP8 confocal microscope (Leica). Live organoids in IF^∗^ media were imaged on a CellDiscoverer 7 (Zeiss).

#### FACS

Mature differentiated hGLU-Venus organoids in IF or IF^∗^ media showing Venus expression were collected in ice cold ADF medium, with 48-96 domes of organoids collected for each experiment. Organoids were then placed in TrypLE (supplemented with 10 μM Y-27632) and digested for 20 min at 37°C before mechanical disruption with a P200/P20 pipette, and then placed in fresh TrypLE for a further 10-20 min until organoids were digested to single cells. Cells were then washed several times in ice cold FACS medium (Hanks’ Balanced Salt Solution (without Ca^2+^ or Mg^2+^, Sigma-Aldrich) supplemented with 10% FBS and 10 μM Y-27632) and passed through a 50 μm strainer to remove any remaining cell clusters. Single cells were stained with 2 μg/ml DAPI (Sigma-Aldrich) and 5 μM DRAQ5 (BD Biosciences) for 5 min and then washed several times in FACS medium. Cells were sorted using a BD FACSMelody cell sorter, forward scatter (FSC) and side scatter (SSC) used to distinguish single cells. Live cells were identified as DAPI negative and DRAQ5 positive populations. Venus intensity was used to sort positive (fluorescent) and negative populations directly into 350 μl RLT+ buffer (QIAGEN) supplemented with 1% b-mercaptoethanol (1-16 × 10^3^ Venus positive cells collected per sort) for RNA sequencing or 250 μl 6M guanidine hydrochloride for mass spectrometry (11-26 × 10^3^ cells per sort).

#### Library preparation for RNA sequencing

RNA extraction and isolation were performed immediately after FAC sorting using RNAeasy Micro Plus kit (QIAGEN). RNA concentration and quality (RIN 6-9) were assessed using RNA6000 Pico Kit and Bioanalyser 2100 (Agilent). cDNA libraries were generated from 2 ng input RNA per sample using the SMARTer Stranded Total RNA-Seq v2 Pico Input Mammalian kit (Takara Bio), with thirteen PCR cycles used for amplification. Libraries were pooled and paired-end 50 bases sequenced on a NovaSeq 6000 (Illumina).

#### Generation of 2D organoid monolayer cultures

To facilitate solution exchange during secretion, electrophysiology, and calcium imaging experiments, two-dimensional (2D) monolayer cultures were derived from well-established hGLU-Venus organoids in IF or IF^∗^ media showing Venus fluorescence. Organoids were collected in ice cold ADF medium and centrifuged at 400 g for 5 min. The resulting pellet was enzymatically digested in TrypLE for 8-10 min at 37°C, further mechanically broken up into small cell clusters and neutralized with 10% FBS in ADF. After centrifugation at 400 g for 5 min, the pellet was resuspended in IF media (supplemented with 10 μM Y-27632) and seeded onto 2% Matrigel (Corning) pre-coated 24-well plates (secretion assays), 35 mm glass-bottom dishes (MatTek, calcium imaging) or 35 mm plastic dishes (electrophysiology) and incubated at 37°C (5% CO_2_) for 18-72 hours prior to experiments.

#### Secretion assays

Saline buffer used for secretion, calcium imaging, and electrophysiology experiments contained 138 mM NaCl, 4.5 mM KCl, 4.2 mM NaHCO_3_, 1.2 mM NaH_2_PO_4_, 2.6 mM CaCl_2_, 1.2 mM MgCl_2_, 10 mM HEPES; adjusted to pH 7.4 with NaOH.

Following overnight incubation, 2D cultures were washed three times in warm saline buffer and incubated for 30 min at 37°C. Buffer was then completely removed before test reagents, dissolved in 200 μl saline buffer supplemented with 10 mM glucose (except where otherwise stated) and fatty acid-free bovine serum albumin (0.1% for GLP-1 immunoassay measurements or 0.001% for mass spectrometry), were added to cultures. After incubation at 37°C for one (mass spectrometry) or two hours (immunoassay), secretion supernatants were collected and centrifuged at 2000 g for five min at 4°C to pellet any debris. The resulting supernatant was transferred to a new tube and snap frozen prior to analysis. Total GLP-1 was measured using a total GLP-1 immunoassay (MesoScale Discovery). For mass spectrometry, samples from three wells were combined and collected in Lobind tubes (Eppendorf).

#### Electrophysiology

Following overnight incubation, 2D cultures were washed with saline buffer supplemented with 0.1mM glucose. Experiments were performed on fluorescent single cells at room temperature (20-24°C). Drugs were applied directly onto cells using a custom-made gravity fed perfusion system. A constant flow of external solution (saline buffer supplemented with 0.1mM glucose) was applied onto cells during baseline recordings and switched to a drug solution during drug applications to avoid flow-induced artifacts.

The internal pipette solution contained: 76 mM K_2_SO_4_, 10 mM NaCl, 10 mM KCl, 10 mM HEPES, 55 mM sucrose, 1 mM MgCl_2_; adjusted to pH 7.2 with KOH. Amphotericin B (10 μg/ml) dissolved in DMSO was added to the pipette solution on the day of recording.

Membrane potential was recorded in the perforated-patch configuration using an Axopatch 200B amplifier connected through a Digidata 1440A A/D converter and pCLAMP software (Molecular Devices). Microelectrodes were pulled from borosilicate glass (1.5 mm OD, 1.16 mm ID; Harvard Apparatus) and the tips coated with refined yellow beeswax. Electrodes were fire-polished using a microforge (Narishige) and had resistances of 2-3 MΩ when filled with pipette solution. A silver/AgCl ground wire connected to the bath solution via a 0.15 M NaCl agar bridge was used as a ground.

Spontaneous action potential firing was recorded in current-clamp mode without injecting current (I = 0). To trigger action potential firing, current was injected to maintain the cell at −75 mV and 10 ms current pulses, increasing in magnitude by 2 pA, were applied at 0.2 Hz. A protocol with longer current injection pulses (500 ms), increasing in magnitude by 2 pA, was also applied to assess the pattern of action potential firing.

#### Calcium imaging

Experiments were performed on fluorescent cells at room temperature using a custom-made gravity-assisted perfusion system as for electrophysiology. Cultures were loaded with 5 μM Fura2-AM (15 min at 37°C then 15 min at room temperature). Fura2 excitation ratio (340/380 nm) was measured using a 40X oil objective (1.35NA) on an inverted microscope (Olympus IX71) with Metafluor software (Molecular Devices), capturing images every 2 s.

#### Peptide characterization by liquid chromatography mass spectrometry

Secretion samples were thawed on ice and 50 μl of 1% formic acid (FA) in water (v/v) was added to each sample prior to transfer onto an Oasis HLB μElution solid phase extraction (SPE) plate (Waters) and extracted on a positive pressure SPE manifold (Waters). All samples were washed with 200 μl 0.1% FA in water (v/v) followed by 200 μl 5% methanol:1% acetic acid in water (v/v). Samples were eluted into a clean low bind plate (QuanRecovery, Waters), with two 30 μl aliquots of 60% methanol:10% acetic acid in water (v/v).

Differentiated organoid cultures (4-24 domes) were collected in cold ADF, washed with cold PBS (3 × 5 min) and lysed for analysis by the addition of 6M guanidine hydrochloride. Samples were precipitated with 1:5 ratio of 80% acetonitrile (ACN) in water, centrifuged at 3500 g for 5 min at 4°C and the aqueous phase (lower), containing the peptides, was transferred to a clean Lobind tube. The supernatant was evaporated under nitrogen at 40°C on a Biotage SPE dry system (Uppsala, Sweden), and reconstituted in 250 μl 0.1% FA in water (v/v) for SPE, as described previously. The SPE eluant was evaporated and reconstituted into 75 μl of 10 mM DTT in 50mM ammonium bicarbonate for reduction. The samples were incubated for 1 hour at 60°C and then alkylated with 20 μl 100 mM iodoacetamide in 50 mM ammonium bicarbonate and incubated for 30 min at room temperature in the dark.

Sorted cells were subjected to three freeze thaw events before 900 μl of 80% ACN in water was added and mixed thoroughly by vortexing. The aqueous layer (lower) was removed and evaporated in a rotary evaporator (Eppendorf) for 18 hours. The residue was reconstituted with 500 μl of 0.1% FA in water (v/v) then extracted by SPE, evaporated, reduced, and alkylated, as previously described, prior to LC-MS analysis.

Prior to injection onto the liquid chromatography (LC) system, samples were diluted with 75 μl 0.1% FA (secretion supernatants) or 25 μl 1% FA (lysed organoids and sorted cells) in water (v/v) and centrifuged at 3500 g for 10 min. Peptide extracts were analyzed using a Thermo Fisher Ultimate 3000 Nano LC system coupled to a Q Exactive Plus Orbitrap mass spectrometer (Thermo Scientific, San Jose, CA, USA). Samples (30 μl for secretion and lysed organoids, and 40 μl for sorted cells) were loaded onto a 0.3 × 5 mm peptide trap column (Thermo Scientific) at a flow rate of 30 μl/min and washed for 15 min before switching in line with a 0.075 × 250 mm nano easy column (Thermo Scientific) flowing at 300 nl/min. Both nano and trap column temperatures were set at 45°C. The mobile phases were A: 0.1% FA in water (v/v) and B: 0.1% FA (v/v) in 80:20 ACN:water. Initial conditions were 2.5% B and held for 15 mins. A ramp to 50% B was performed over 90 min, and the column then washed with 90% B for 20 min before returning to starting conditions for a further 20 mins, totaling an entire run time of 130 min. Positive nano electrospray analysis was performed using a spray voltage of 1.8 kV, and an S-lens setting of 70 V. A full scan range of 400–1600 m/z was performed at a resolution of 75,000 before the top 10 ions of each spectrum were selected for MS/MS analysis. Existing ions selected for fragmentation were added to an exclusion list for 30 s.

### Quantification and Statistical Analysis

Statistical analysis of individual experiments is further detailed in the figure legends.

#### RNA sequencing analysis

Quality and adaptor trimming of sequenced transcripts was performed using cutadapt (v2.7). BBMap (v38.76) was used to align transcripts to the human genome (GRCh38). Raw counts were generated using featureCounts (v2.0.0). Quality controls were performed after each processing step using FastQC (v0.11.9). Differential gene expression analysis was performed in RStudio using DESeq2 (v1.24.0). Gene annotation was obtained from the Ensembl dataset held in BioMart (v2.40.5). Receptor and ion channel lists were generated from the International Union of Basic and Clinical Pharmacology (IUPHAR)/British Pharmacological Society (BPS) “targets and families” list (Accessed on 7 Jan 2020).

#### GLP-1 secretion assays

GLP-1 secretion is expressed as fold change in supernatant GLP-1 content, relative to the mean level of basal (10mM glucose, except for glucose sensing experiments which are expressed relative to 0mM glucose) samples performed in parallel. Data were tested for normality with a Shapiro-Wilk test and log transformed if necessary. Secretion conditions were compared by two-way or three-way analysis of variance with Tukey honest significant difference post hoc tests.

#### Calcium imaging analysis

Mean whole cell Fura2-AM ratios (340nm/380nm) were calculated following background subtraction (Metafluor). Fold change in maximum ratio before and during treatment (F/F_0_) were calculated and analyzed by one-sample two-tailed Student’s t test.

#### Electrophysiology analysis

The inter-spike membrane potential (ISMP) was assessed by plotting an “all-points” histogram of a 30 s recording before or during treatment; the mode was taken as ISMP. To assess action potential firing frequency, a threshold of −10 mV was used to positively identify action potentials within a 60 s analysis window, and action potential firing frequency was expressed as number of action potentials fired per s (Hz). To measure action potential waveform properties, evoked responses from short current injections (10 ms) were used. The action potential peak was taken as the maximum voltage reached and threshold determined as the voltage at which the upstroke in voltage begins. The width of the action potential waveform was measured at 50% of the action potential peak, or the action potential half-width. To evaluate the pattern of action potential firing, the total number of action potentials evoked during longer current injection pulses (500 ms) that crossed a threshold of −10 mV was plotted against the magnitude of the current injected.

To determine if drug responses were statistically significant, a one-sample t test, compared to a theoretical mean of 0, was applied, except for evaluating the effect of GPBAR-A over baseline on the pattern of action potential firing where multiple t tests were performed for each current injection pulse. To compare three or more groups, a Kruskal-Wallis with Dunn’s multiple comparisons test was used.

#### Immunohistochemistry analysis

Analysis of immunohistochemistry images was performed in ImageJ with Fiji. Co-localization of proGCG and Venus (GFP) staining was analyzed using the Cell Counter plugin. Mean whole cell intensity of proGCG and Venus staining were measured and Pearson correlation coefficient calculated.

#### LC-MS analysis

Peptidomic searches of the human Uniprot database (downloaded October 2018) were performed using PEAKS (v8.5, BSI) and the peak integration was performed using Xcalibur (v4.3.73.11, ThermoFisher Scientific). Peak area intensity of parental proteins in sorted cells was calculated in PEAKS. Peak area of secreted peptides was compared between basal and stimulated conditions by multiple paired two-sample two-tailed Student’s t tests.

## Supplemental Information

Document S1. Figure S1

Document S2. Article plus Supplemental Information

## References

[bib1] Basak O., Beumer J., Wiebrands K., Seno H., van Oudenaarden A., Clevers H. (2017). Induced quiescence of Lgr^5+^ stem cells in intestinal organoids enables differentiation of hormone-producing enteroendocrine cells. Cell Stem Cell.

[bib2] Beumer J., Artegiani B., Post Y., Reimann F., Gribble F., Nguyen T.N., Zeng H., Van den Born M., Van Es J.H., Clevers H. (2018). Enteroendocrine cells switch hormone expression along the crypt-to-villus BMP signalling gradient. Nat. Cell Biol..

[bib3] Capozzi M.E., DiMarchi R.D., Tschöp M.H., Finan B., Campbell J.E. (2018). Targeting the incretin/glucagon system with triagonists to treat diabetes. Endocr. Rev..

[bib4] Dye F.S., Larraufie P., Kay R., Darwish T., Rievaj J., Goldspink D.A., Meek C.L., Middleton S.J., Hardwick R.H., Roberts G.P. (2019). Characterisation of proguanylin expressing cells in the intestine—evidence for constitutive luminal secretion. Sci. Rep..

[bib5] Frias J.P., Nauck M.A., Van J., Kutner M.E., Cui X., Benson C., Urva S., Gimeno R.E., Milicevic Z., Robins D., Haupt A. (2018). Efficacy and safety of LY3298176, a novel dual GIP and GLP-1 receptor agonist, in patients with type 2 diabetes: a randomised, placebo-controlled and active comparator-controlled phase 2 trial. Lancet.

[bib6] Fujii M., Matano M., Nanki K., Sato T. (2015). Efficient genetic engineering of human intestinal organoids using electroporation. Nat. Protoc..

[bib7] Fujii M., Matano M., Toshimitsu K., Takano A., Mikami Y., Nishikori S., Sugimoto S., Sato T. (2018). Human intestinal organoids maintain self-renewal capacity and cellular diversity in niche-inspired culture condition. Cell Stem Cell.

[bib8] Gilon P., Chae H.Y., Rutter G.A., Ravier M.A. (2014). Calcium signaling in pancreatic β-cells in health and in Type 2 diabetes. Cell Calcium.

[bib9] Goldspink D.A., Matthews Z.J., Lund E.K., Wileman T., Mogensen M.M. (2017). Immuno-fluorescent labeling of microtubules and centrosomal proteins in ex vivo intestinal tissue and 3D in vitro intestinal organoids. J. Vis. Exp..

[bib10] Goldspink D.A., Lu V.B., Billing L.J., Larraufie P., Tolhurst G., Gribble F.M., Reimann F. (2018). Mechanistic insights into the detection of free fatty and bile acids by ileal glucagon-like peptide-1 secreting cells. Mol. Metab..

[bib11] Gorboulev V., Schürmann A., Vallon V., Kipp H., Jaschke A., Klessen D., Friedrich A., Scherneck S., Rieg T., Cunard R. (2012). Na^+^-D-glucose cotransporter SGLT1 is pivotal for intestinal glucose absorption and glucose-dependent incretin secretion. Diabetes.

[bib12] Gribble F.M., Reimann F. (2016). Enteroendocrine cells: chemosensors in the intestinal epithelium. Annu. Rev. Physiol..

[bib13] Gribble F.M., Reimann F. (2019). Function and mechanisms of enteroendocrine cells and gut hormones in metabolism. Nat. Rev. Endocrinol..

[bib14] Gribble F.M., Diakogiannaki E., Reimann F. (2017). Gut hormone regulation and secretion via FFA1 and FFA4. Handb. Exp. Pharmacol..

[bib15] Habib A.M., Richards P., Rogers G.J., Reimann F., Gribble F.M. (2013). Co-localisation and secretion of glucagon-like peptide 1 and peptide YY from primary cultured human L cells. Diabetologia.

[bib16] Holst J.J. (2020). Incretin therapy for diabetes mellitus type 2. Curr. Opin. Endocrinol. Diabetes Obes..

[bib17] Kuhre R.E., Frost C.R., Svendsen B., Holst J.J. (2015). Molecular mechanisms of glucose-stimulated GLP-1 secretion from perfused rat small intestine. Diabetes.

[bib18] Liao Y., Smyth G.K., Shi W. (2014). featureCounts: an efficient general purpose program for assigning sequence reads to genomic features. Bioinformatics.

[bib19] Love M.I., Huber W., Anders S. (2014). Moderated estimation of fold change and dispersion for RNA-seq data with DESeq2. Genome Biol..

[bib20] Ma J., Bellon M., Wishart J.M., Young R., Blackshaw L.A., Jones K.L., Horowitz M., Rayner C.K. (2009). Effect of the artificial sweetener, sucralose, on gastric emptying and incretin hormone release in healthy subjects. Am. J. Physiol. Gastrointest. Liver Physiol..

[bib21] Martin M. (2011). Cutadapt removes adapter sequences from high-throughput sequencing reads. EMBnet J..

[bib22] Müller T.D., Finan B., Bloom S.R., D’Alessio D., Drucker D.J., Flatt P.R., Fritsche A., Gribble F., Grill H.J., Habener J.F. (2019). Glucagon-like peptide 1 (GLP-1). Mol. Metab..

[bib23] Nielsen L.B., Ploug K.B., Swift P., Ørskov C., Jansen-Olesen I., Chiarelli F., Holst J.J., Hougaard P., Pörksen S., Holl R., Hvidøre Study Group (2007). Co-localisation of the Kir6.2/SUR1 channel complex with glucagon-like peptide-1 and glucose-dependent insulinotrophic polypeptide expression in human ileal cells and implications for glycaemic control in new onset type 1 diabetes. Eur. J. Endocrinol..

[bib24] Pais R., Rievaj J., Larraufie P., Gribble F., Reimann F. (2016). Angiotensin II type 1 receptor-dependent GLP-1 and PYY secretion in mice and humans. Endocrinology.

[bib25] Pais R., Rievaj J., Meek C., De Costa G., Jayamaha S., Alexander R.T., Reimann F., Gribble F. (2016). Role of enteroendocrine L-cells in arginine vasopressin-mediated inhibition of colonic anion secretion. J. Physiol..

[bib26] Parker V.E.R., Robertson D., Wang T., Hornigold D.C., Petrone M., Cooper A.T., Posch M.G., Heise T., Plum-Moerschel L., Schlichthaar H. (2020). Efficacy, safety, and mechanistic insights of cotadutide, a dual receptor glucagon-like peptide-1 and glucagon agonist. J. Clin. Endocrinol. Metab..

[bib27] Ran F.A., Hsu P.D., Wright J., Agarwala V., Scott D.A., Zhang F. (2013). Genome engineering using the CRISPR-Cas9 system. Nat. Protoc..

[bib28] Roberts G.P., Kay R.G., Howard J., Hardwick R.H., Reimann F., Gribble F.M. (2018). Gastrectomy with Roux-en-Y reconstruction as a lean model of bariatric surgery. Surg. Obes. Relat. Dis..

[bib29] Roberts G.P., Larraufie P., Richards P., Kay R.G., Galvin S.G., Miedzybrodzka E.L., Leiter A., Li H.J., Glass L.L., Ma M.K.L. (2019). Comparison of human and murine enteroendocrine cells by transcriptomic and peptidomic profiling. Diabetes.

[bib30] Rogers G.J., Tolhurst G., Ramzan A., Habib A.M., Parker H.E., Gribble F.M., Reimann F. (2011). Electrical activity-triggered glucagon-like peptide-1 secretion from primary murine L-cells. J. Physiol..

[bib31] Sato T., Vries R.G., Snippert H.J., van de Wetering M., Barker N., Stange D.E., van Es J.H., Abo A., Kujala P., Peters P.J., Clevers H. (2009). Single Lgr5 stem cells build crypt-villus structures in vitro without a mesenchymal niche. Nature.

[bib32] Sato T., Stange D.E., Ferrante M., Vries R.G., Van Es J.H., Van den Brink S., Van Houdt W.J., Pronk A., Van Gorp J., Siersema P.D., Clevers H. (2011). Long-term expansion of epithelial organoids from human colon, adenoma, adenocarcinoma, and Barrett’s epithelium. Gastroenterology.

[bib33] Schindelin J., Arganda-Carreras I., Frise E., Kaynig V., Longair M., Pietzsch T., Preibisch S., Rueden C., Saalfeld S., Schmid B. (2012). Fiji: an open-source platform for biological-image analysis. Nat. Methods.

[bib34] Shah B.P., Liu P., Yu T., Hansen D.R., Gilbertson T.A. (2012). TRPM5 is critical for linoleic acid-induced CCK secretion from the enteroendocrine cell line, STC-1. Am. J. Physiol. Cell Physiol..

[bib35] Sjölund K., Sandén G., Håkanson R., Sundler F. (1983). Endocrine cells in human intestine: an immunocytochemical study. Gastroenterology.

[bib36] Sun E.W., de Fontgalland D., Rabbitt P., Hollington P., Sposato L., Due S.L., Wattchow D.A., Rayner C.K., Deane A.M., Young R.L., Keating D.J. (2017). Mechanisms controlling glucose-induced GLP-1 secretion in human small intestine. Diabetes.

[bib37] Wu T., Zhao B.R., Bound M.J., Checklin H.L., Bellon M., Little T.J., Young R.L., Jones K.L., Horowitz M., Rayner C.K. (2012). Effects of different sweet preloads on incretin hormone secretion, gastric emptying, and postprandial glycemia in healthy humans. Am. J. Clin. Nutr..

