# Supplementary material for: Labeling and Characterization of Human GLP-1-Secreting L-cells in Primary Ileal Organoid Culture
Source: Cell Rep. 2020 Jun 30;31(13):107833. doi: 10.1016/j.celrep.2020.107833 (PMC7342002; doi:10.1016/j.celrep.2020.107833)
Supplement: Document S1. Figure S1 [file mmc1.pdf]

Cell Reports, Volume *31*

Supplemental Information

Labeling and Characterization of Human GLP-1-Secreting L-cells in Primary Ileal Organoid Culture

Deborah A. Goldspink, Van B. Lu, Emily L. Miedzybrodzka, Christopher A. Smith, Rachel E. Foreman, Lawrence J. Billing, Richard G. Kay, Frank Reimann, and Fiona M. Gribble

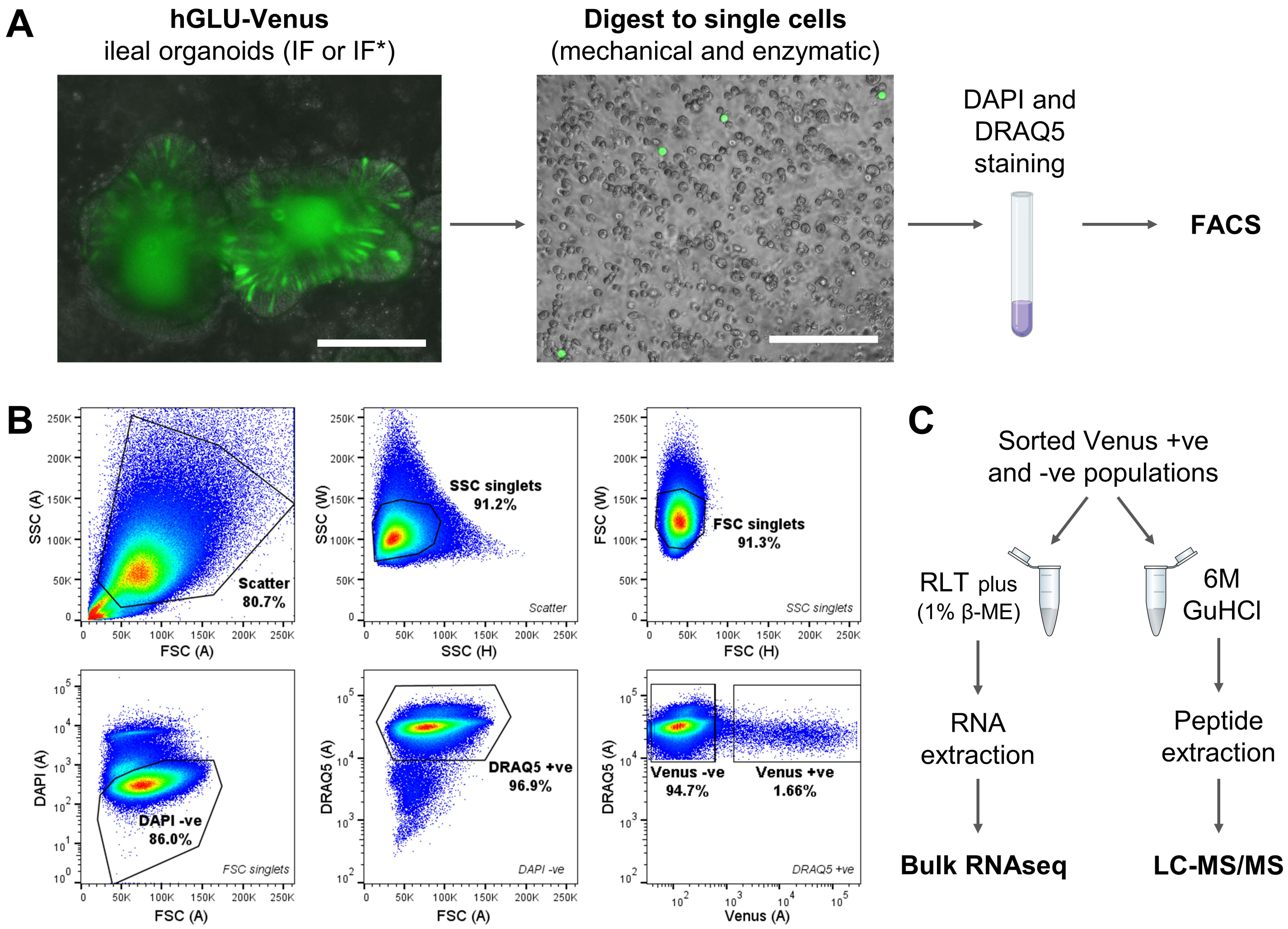
A
hGLU-Venus
ileal organoids (IF or IF*)
Digest to single cells
(mechanical and enzymatic)
DAPI and
DRAQ5
staining
FACS
B
SSC (A)
FSC (A)
Scatter
80.7%
SSC (W)
SSC (H)
SSC singlets
91.2%
Scatter
FSC (W)
FSC (H)
FSC singlets
91.3%
SSC singlets
DAPI (A)
FSC (A)
DAPI -ve
86.0%
FSC singlets
DRAQ5 (A)
FSC (A)
DRAQ5 +ve
96.9%
DAPI -ve
DRAQ5 (A)
Venus (A)
Venus -ve
94.7%
Venus +ve
1.66%
DRAQ5 +ve
C
Sorted Venus +ve
and -ve populations
RLT plus
(1% β-ME)
6M
GuHCl
RNA
extraction
Peptide
extraction
Bulk RNAseq
LC-MS/MS

Figure S1: FACS purification of hGLU-Venus positive cells (related to Figure 2)

A. hGLU-Venus organoids cultured in either IF or IF* medium (example organoid shown in IF* medium) were digested to single cells (scale bars 200 μm), incubated with DRAQ5 and DAPI and FACsorted. B. Single cells were selected based on their side scatter (SSC) and forward scatter (FSC) (*top row*) and live single cells were further isolated from dead cells and debris based on DAPI and DRAQ5 fluorescence as indicated, before Venus-fluorescent cells were distinguished from non-fluorescent cells (*bottom row*). In the example shown, 1.66% of the live single cell population were Venus-positive. C. Both Venus-positive and Venus negative cells were sorted separately either directly into lysis buffer for RNA extraction or alternatively into guanidine hydrochloride (GuHCl) for subsequent LC-MS/MS analysis. FACS plots represent the results from 500k events.
